# Relative Deprivation and Hope: Predictors of Risk Behavior

**DOI:** 10.1007/s10899-020-09989-4

**Published:** 2020-12-16

**Authors:** Shahriar Keshavarz, Kenny R. Coventry, Piers Fleming

**Affiliations:** School of Psychology, University of East Anglia, Norwich, NR4 7TJ UK

**Keywords:** Relative deprivation, Hope, Gambling, Risk, Intervention

## Abstract

The belief that one is in a worse situation than similar others (Relative Deprivation) has been associated with involvement in a range of maladaptive escape behaviors, including excessive risk taking. Yet not everyone scoring high on measures of relative deprivation makes maladaptive choices. We hypothesized that *hope* may ameliorate the negative effects of relative deprivation. In two laboratory-based experiments using a novel risk-taking task (*N* = 101) we show that hope reduces risk-taking behavior in relatively deprived participants. A third study (*N* = 122) extended the moderating effect of hope on relative deprivation to real-world risk behavior; increased hope was associated with decreased likelihood of loss of control of one’s gambling behavior in relatively deprived individuals. Nurturing hope in relatively deprived populations may protect them against maladaptive behaviors with potential applications for harm reduction.

## Introduction

“Comparison is the thief of joy.”

Theodore Roosevelt

The concept of Relative Deprivation (RD) has been widely adopted throughout the social sciences - from criminology (e.g., Lea and Young 1993) and economics (e.g., Yitzhaki 1979) to political science (Lichbach 1990) and history (Snyder and Tilly 1972) - to characterize the comparison between an individual and others. Operationally, Relative Deprivation (RD) is the belief that one is in a worse situation than similar others (e.g., neighbours), an observation that triggers negative emotions such as anger and resentment (see Crosby 1976; Folger 1987; Stiles et al. 2000), which in turn triggers achievement, escape and/or deviant behaviors (see Smith et al. 2012 for a review). Thus, RD is a social psychological construct; “it postulates a subjective state that shapes emotions, cognitions and behavior” (Pettigrew 2016, *p.* 9), and is a key concept invoked to explain a range of behaviors across the social sciences (Walker and Smith 2002).

The feelings of anger and resentment stemming from RD encourage the relatively deprived to engage in behaviors that allay or overcome negative emotions (Smith et al. 2012). RD has been directly associated with a range of addictive behaviors, including alcohol consumption and excessive gambling (see Table 1). For instance, Callan et al. (2011) reported that “because personal relative deprivation is an aversive state, people are often motivated to reduce it by engaging in various [risky] behaviors” (*p*. 956). Yet it is also the case that not all relatively deprived individuals engage in maladaptive behaviors. One promising construct that we hypothesise may moderate the relationship between RD and maladaptive behavior is the psychological construct of *hope*. Hope is “a positive motivational state that is based on an interactively derived sense of successful (a) agency (goal-directed energy), and (b) pathways (planning to meet goals)” (Snyder et al. 1991, *p*. 287). The pathway component of hope reflects the ability to produce plausible alternate routes when pursuing desired goals (Snyder 2002). Agency-thinking represents the motivational component of hope theory, that is, the mental energy that is required to pursue goals (Snyder 2002). Although agency-thinking is important in all aspects of goal pursuit, it is especially vital when encountering obstacles (Snyder 1994, 2002).

**Table 1:** Relative deprivation as a predictor of deviant, escape and achievement behaviors

		Author and Year	Findings
Negative	Deviant behaviors	Napoletano et al. (2016)	RD is positively related to two types of bullying perpetration (relational and cyber)
		Stiles et al. (2000)	RD induces negative feelings, which in turn motivate property crimes and violence
		Helgertz et al. (2013)	As relative income increases, absence from work declines
		Odgers et al. (2015)	Children experiencing RD (i.e., those surrounded by more affluent neighbors) engage in more antisocial behaviors than their peers living in concentrated poverty
	Escape behaviors	Horne (2009)	RD is positively related to alcohol and marijuana use among juveniles
		Eibner and Evans (2005)	Higher RD is associated with a higher probability of smoking
		Callan et al. (2008)	RD is positively associated with desires to gamble
		Sim et al. (2018)	RD is positively associated with excess calorie intake
Positive	Achievement behaviors	Turley (2002) and Zoogah (2010)	RD is associated with positive behaviors (e.g., relatively deprived are more self-reliant)
		Wilensky (1963)	Some relatively deprived attempt to improve by working a second job (moonlighting)
		Feldman and Turnley (2004)	RD is positively related to efforts to find alternative (potentially better) employment
		Olson et al. (1995)	RD is predictive of willingness to engage in self-improvement behaviors

Therefore, in the face of RD, we predict that high-hopers are likely to possess the motivational energy (agency-thinking) required to overcome RD. However, low-hopers’ lack of agency-thinking to overcome RD is likely to encourage the use of avoidant-coping strategies (i.e., gambling) that temporarily allay negative feelings that they continue to face. There is much evidence that remaining hopeful in the face of adversity can be advantageous (see Snyder 2002; Valle et al. 2006). For instance, when hopeful individuals face obstacles during goal pursuit, they show flexibility in their approach and find alternative pathways that they persist with (Snyder 2002). In contrast, individuals low in hope ruminate about the hurdle (Michael 2000) and “engage in almost magical escape fantasies” (Snyder 2002, *p*. 261). This also showcases the overlap between hope and executive function abilities such as flexibility, planning and self-control, all of which are vital in dealing with adversities; research has found greater executive function is associated with greater pathways and agency components of hope (see Fallucca 2018; Kruger 2011; Sears 2007). Indeed, the overlap between hope and executive functions further suggests that higher levels of hope can protect the vulnerable against maladaptive behaviors, especially as studies have found a relationship between poor executive functions (i.e., planning) and engagement in problem behaviors (e.g., problem gambling) (von Hippel et al. 2009; Ledgerwood et al. 2012).

To put the above into context, imagine two relatively deprived individuals, Harry and Clive. Harry (a high-hope individual) and Clive (a low-hoper) both believe that participation in career development activities can lead to a better-paid job, which can help overcome RD and the accompanying negative emotions. Both Harry and Clive also know that gambling can temporarily alleviate the negative emotions stemming from RD but is unlikely to help in overcoming RD altogether; instead, losing money is likely to magnify the problem. Nonetheless, Clive is unable to stay motivated in the face of obstacles, instead, Clive ruminates over obstacles and uses gambling to forget about his problems. Harry, not fazed by obstacles, decides to think of several methods in which he can achieve the same outcome, overcoming RD. Even during moments of desperation, Harry remains hopeful and motivated to continue along the path he knows is best. It is this difference in hope that indicates whether an individual is likely to succeed in overcoming adversities (i.e., RD and the accompanying negative emotions) or instead relies on escape behaviors to cope with the negative emotions that they continue to face.

In summary, it is evident that the constructs of RD and hope are individually predictive of a range of activities, but they have not previously been considered together. Below we explore for the first time whether hope moderates participation in a risk-taking context both in the laboratory (Experiments 1 and 2) and in the field (as measured by participation in gambling, Experiment 3).

## Experiment 1

We hypothesized that increases in hope would reduce risk-taking among relatively deprived individuals. To put our hypotheses to the test, Experiment 1 examined whether self-report measures of hope and RD predict risk-taking in a novel gambling-like risk game in the laboratory.

### Methods

#### Participants

Fifty-five participants (45 females and 10 males; age range 18–22 years, *M* = 19.58, *SD* = 1.07) were recruited from the student population on a university campus via a volunteer participant credit system. The sample consists of 82% female and 18% male participants, which is representative of the undergraduate Psychology student population at the university campus where data were collected. A priori power analysis (using the G*Power 3.1 tool: Faul et al. 2009) indicated that for a regression model consisting of 7 predictor variables, 51 participants would be required to detect a large effect (*R*^2^ = 0.25) with 80% power (*1-β err prob* = 0.8).

#### Materials

##### The Adult Hope Scale

Snyder et al.’s (1991) Adult Hope Scale was used to measure participants’ level of hope; the scale has been shown to load reliably on two-factors across languages (e.g., English, French, Japanese and Portuguese) and diverse populations (see Gana et al. 2013; Kato and Snyder 2005; Marques et al. 2014; Snyder et al. 1991). The 12-item scale is divided into two subscales based on Snyder’s cognitive model of hope: (1) Agency (i.e., goal-directed energy) and (2) Pathways (i.e., number of alternate routes to desired goals). Four of the 12 items make up the agency subscale (e.g., *I energetically pursue my goals*), four make up the pathway subscale (e.g., *I can think of many ways to get out of a jam*), while the remaining four were filler items (e.g., *I feel tired most of the time*). Participants were asked to rate each item on an 8-point scale (1 = definitely false—8 = definitely true).

##### Personal Relative Deprivation Scale (PRDS)

Callan et al.’s (2011) 5-item PRDS (α = 0.78) was designed to measure participants’ general beliefs and feelings of their outcomes relative to similar others. Example items include: *I feel deprived when I think about what I have compared to what other people like me have* and *I feel privileged compared to other people like me*. Each item is rated on a 6-point scale (1 = strongly disagree—6 = strongly agree).

##### Risk Game

**Fig. 1:**
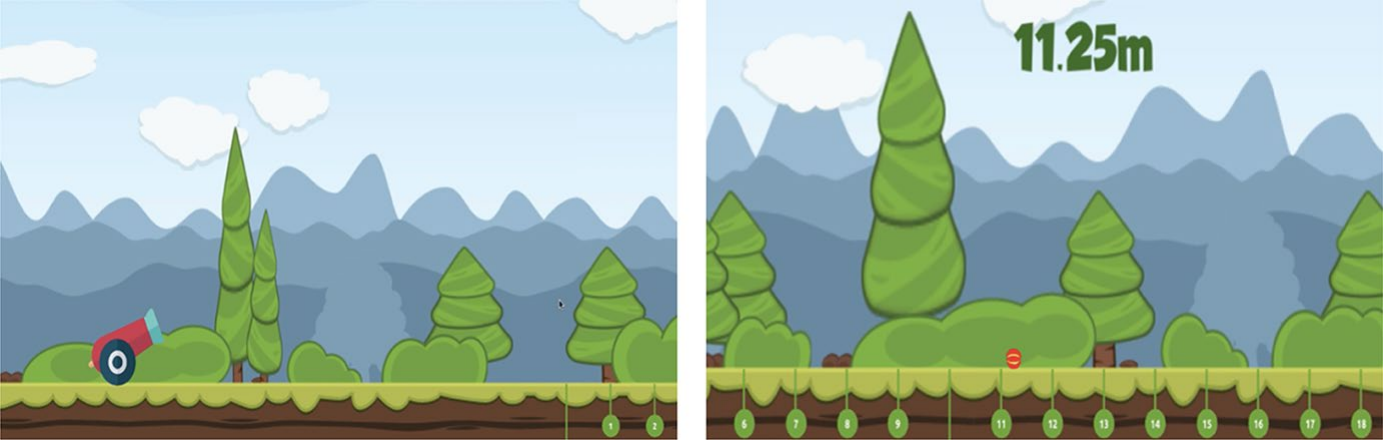
A cannon (left image) fires a ball that could land anywhere between 0 and 100 m from the cannon (11.25 m in this example)

**Fig. 2:**
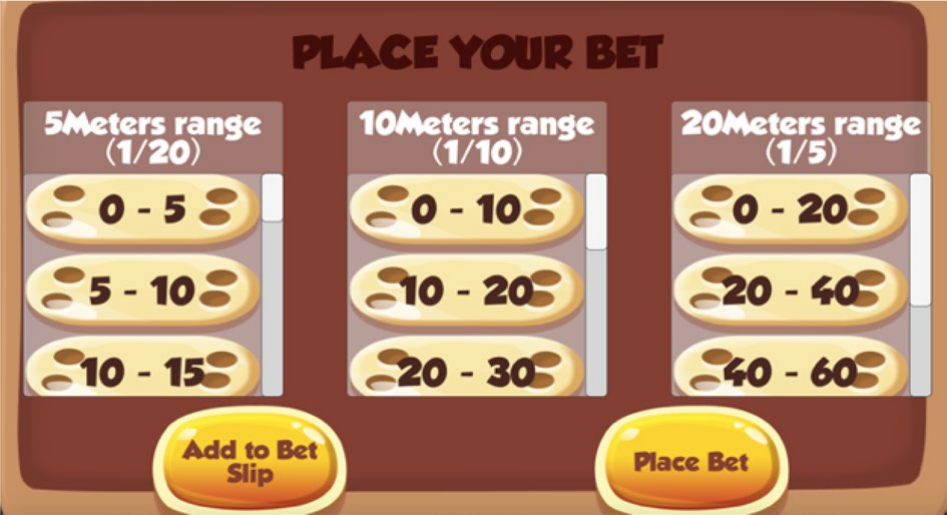
Place your bet page (risk decreasing from left to right)

The task used to measure participants’ risk-taking is a novel game that was created on UNITY software. In the game, a cannon fires a ball that could land anywhere between 0 and 100 m from the cannon (see Fig. 1); participants are asked to bet on where they think the ball will land (see Fig. 2). For instance, a participant may feel that the ball will land between 20 and 30 m, thus covering this ground in their bet. When choosing a distance to cover, participants are given a choice of three range groups to choose from (as shown in Fig. 2): the high-risk option allows participants to cover less ground (5-m range) than both the moderate (10-m range) and low-risk (20-m range) alternatives. However, greater *potential returns* (larger incentives for winning) may make the high-risk option more appealing than the moderate and low-risk alternatives*.* That is, although the low-risk option covers more distance and thus increases one’s chance of winning, the larger incentives (*potential returns*) accompanying the moderate and high-risk options may be more attractive to participants.

The risk game consisted of ten experimental rounds which comprised five loss rounds (rounds 1, 2, 6, 8 and 9); and five win rounds (rounds 3, 4, 5, 7 and 10). Unaware that the game is rigged (until debrief, participants were told that this is a game of chance), in each round, participants were asked to choose a distance range to cover from one of the three risk options. Each bet deducted 10 points from participants’ credit (participants started the game with 100 points in credit—no real money was being bet); participants selected their range from the dropdown menu which also indicated *potential returns* (i.e., points credited for winning). 210 points were credited for winning on the high-risk option, 110 points for winning on the moderate-risk option, and 60 points for winning on the low-risk option, thus higher *potential returns* indicate greater risk-taking. Consequently, the sum of participants’ *potential returns* throughout the ten experimental rounds were used as a measure of risk-taking (higher figures indicate greater risk-taking).

##### Task-Comprehension Questionnaire

A four-item task-comprehension questionnaire was produced to test participants’ understanding of the risk game. Once participants had received a detailed explanation of the risk game and played one trial round, they were asked to complete the first two questions of the task-comprehension questionnaire: (1) how many points could you potentially win by choosing the low-risk option?; and (2) when placing a bet, selecting which of the following odds will lead to a better chance of winning? Participants were able to choose one of the three options given in the risk game (see Fig. 2). After completing questions one and two, feedback was given to participants to ensure that they understood the game. Participants were then asked to complete questions three and four, which were similar to questions one and two; participants were excluded if they failed to answer questions three and four correctly.

#### Procedure

Participants first completed an online questionnaire, prior to attending a laboratory-based session one week later. The online questionnaire comprised demographic questions (age and gender), the Adult Hope scale and the PRDS. During the laboratory-based session, participants initially received a detailed explanation of the risk game and were given one trial round to play, after which, they were asked to complete the task-comprehension questionnaire. Upon completion of the task-comprehension questionnaire, participants were asked to play the risk game for real monetary incentives calculated as £1 for every 200 points gained, though payment was dependent on the rolling of a die. That is, once participants were informed of their score on the risk game, they were asked to choose a number between one to six and to roll a die; payment was made only if the die landed on the participant’s chosen number. The payment structure in this study was due to budgetary/financial constraints. However, research findings indicate that the approach to paying only some participants for their choices does not influence behavior (see Bolle 1990; Charness et al. 2016). Finally, participants were debriefed and thanked for their contribution.

#### Data Analyses

The outcome variable was calculated as the sum of *potential returns* across the ten experimental rounds (greater figures indicate greater risk-taking). A three-stage hierarchical regression model was used to examine whether the relation between RD and risk-taking was moderated by either the agency or pathway components of hope, when controlling for age and gender. The rejection level for all analyses was set at *p* = 0.05.

### Results

**Fig. 3:**
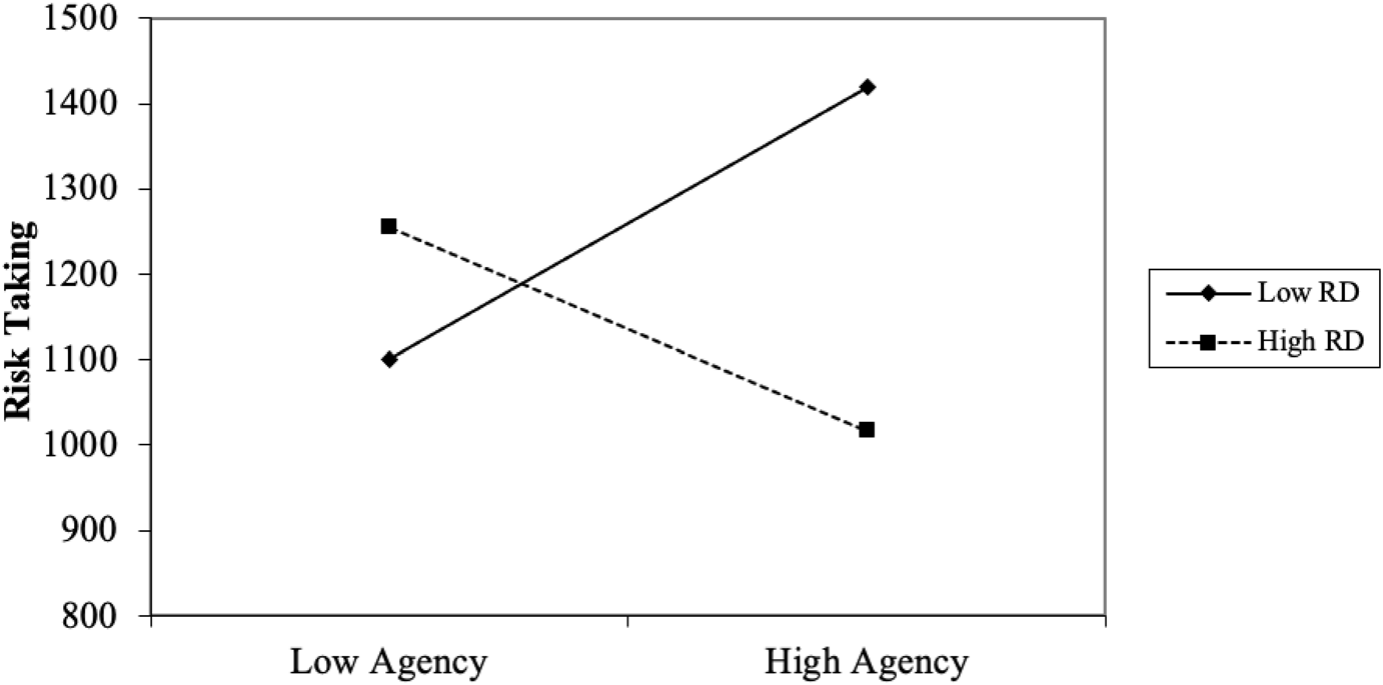
The effect of agency-thinking and RD (in interaction) on risk-taking in Experiment 1. Note. Points are plotted at ± 1 *SD* of the mean

Four participants failed the task-comprehension questionnaire and were thus excluded from analyses. The remaining fifty-one (43 females and 8 males) participants’ data were used for analyses; participants’ ages ranged from 18 to 22 years old (*M* = 19.45, *SD* = 0.97). A three-stage hierarchical regression was conducted with the sum of potential returns as the outcome variable (labelled *risk-taking* from this point on). The covariate variables age and gender were entered at stage one of the regression model as research has shown these to influence gambling behavior (see Johansson et al. 2009). The predictor variables agency, pathway and RD were entered at stage two of the regression and two interaction variables (Agency x RD and Pathway x RD) were computed and entered at stage three. Significant results indicate that the agency component of hope played a moderating role in the relation between RD and risk-taking (see Table 2 and Fig. 3).

**Table 2:** Summary of hierarchical regression analysis for variables predicting risk-taking

Variable	*β*	*95% CI (LL, UL)*	*t*	*SE*	*R*	*R*^*2*^	*ΔR*^*2*^	*F*	*ΔF*	*ΔF p*	*p*
**Model 1**					**0.34**	**0.12**	**0.12**	**3.15**	**3.15**	**0.052**	**0.052**
Age	0.30	(5.64, 169.78)	2.15	40.82							0.037*
Gender	0.12	(−123.40, 308.15)	0.86	107.32							0.394
**Model 2**					**0.37**	**0.14**	**0.02**	**1.40**	**0.33**	**0.804**	**0.241**
Age	0.32	(7.97, 178.59)	2.20	42.35							0.033*
Gender	0.14	(−113.59, 335.06)	0.99	111.38							0.325
Agency	− 0.01	(−23.92, 23.23)	− 0.03	11.71							0.977
Pathway	− 0.15	(−30.44, 11.58)	− 0.90	10.43							0.371
RD	− 0.04	(−26.59, 20.29)	− 0.27	11.64							0.788
**Model 3**					**0.61**	**0.37**	**0.24**	**3.60**	**8.00**	0.001***	0.004**
Age	0.39	(38.09, 189.33)	3.03	37.50							0.004**
Gender	0.11	(−111.81, 285.82)	0.88	98.59							0.382
Agency	0.07	(−16.71, 26.38)	0.45	10.68							0.653
Pathway	− 0.27	(−37.08, 1.80)	− 1.83	9.64							0.074
RD	− 0.22	(−37.97, 5.71)	− 1.49	10.83							0.144
Agency X RD	− 0.51	(−14.26, − 3.28)	− 3.22	2.72							0.002**
Pathway X RD	− 0.04	(−6.30, 4.76)	− 0.28	2.74							0.780

*Note. N* = 51; *p < 0.05; **p < 0.01; ***p < 0.001 *LL* and *UL* indicate the lower and upper limit of a Confidence Interval (for *B*); A post hoc power analysis indicated that our results produced a power of 97.7%, (*1-β err prob* = 0.977) indicating that this study had an adequate sample size

### Discussion

Our results indicate that agency-thinking (i.e., goal-directed energy) is positively correlated with risk-taking when RD scores are low (i.e., when feeling relatively privileged) and negatively correlated with risk-taking when RD scores are high (i.e., when feeling relatively deprived). However, our results show that pathway-thinking (i.e., number of plausible alternate routes to desired goals) and RD, in interaction, do not predict changes in risk-taking. Therefore Experiment 1 provides correlational evidence in support of our hypotheses, but it does not determine whether feelings of relative deprivation *cause* changes in risk-taking among low and high-hope individuals.

## Experiment 2

To test the causal effect of relative deprivation on risk-taking moderated by hope this study employed an experimental manipulation to induce feelings of relative deprivation and privilege. This experimental manipulation was a modified version of that validated by Callan et al. (2008). In short, the aim of this study was to test whether feeling relatively deprived—elicited by the knowledge that one has *less* discretionary income than similar others—causes greater risk-taking among low-hope persons and decreased risk-taking among high-hope persons. As in Experiment 1, age and gender were added as covariates. Moreover, family income was also added as a covariate as participants’ Socioeconomic Status (SES) (family income is one measure of SES) may impact whether the experimental manipulations had the desired effect or not (i.e., individuals with high family income may not believe the cover story used to induce feelings of relative deprivation).

### Methods

#### Participants

Fifty-one participants (41 females and 10 males) were initially recruited from the student population on a university campus. The sample, which consists of a majority of female participants, is representative of the undergraduate Psychology student population at the university campus where data were collected. All 51 students were Psychology students recruited via a volunteer participant credit system; participants’ age ranged from 18 to 39 years old (*M* = 20.31, *SD* = 3.28). While a priori power analysis using the G*Power 3.1 tool (Faul et al. 2009) indicated that a regression model consisting of 8 predictor variables would require 54 participants to detect a large effect (*R*^2^ = 0.25) with 80% power (*1-β err prob* = 0.8), post hoc power analysis from Experiment 1 indicated that a sample size of 51 will produce a high statistical power.

#### Materials and Procedure

Participants first completed an online questionnaire before attending a laboratory-based session a week later. The online questionnaire comprised the Adult Hope scale (as in Experiment 1), as well as demographic and personal information questions (i.e., age, gender, home postcode and family income). During the laboratory session, to deceive participants into believing that they are relatively deprived/privileged in comparison to similar others, and thus induce feelings of relative deprivation/privilege, we formulated the cover story that answers provided in the online questionnaire were used to calculate Comparative Discretionary Income (CDI) index scores. We used the following text to induce feelings of relative deprivation/privilege:

Your Comparative Discretionary Income (CDI) index score was derived from statistical analyses using both the information from the online questionnaire you completed and the information in our database from people who matched your profile. From your CDI index score, we were able to figure out how deprived/privileged you are in comparison to similar others (i.e., similar age, gender, etc.). The scale below points to the range your CDI index score falls within.

**Fig. 4:**
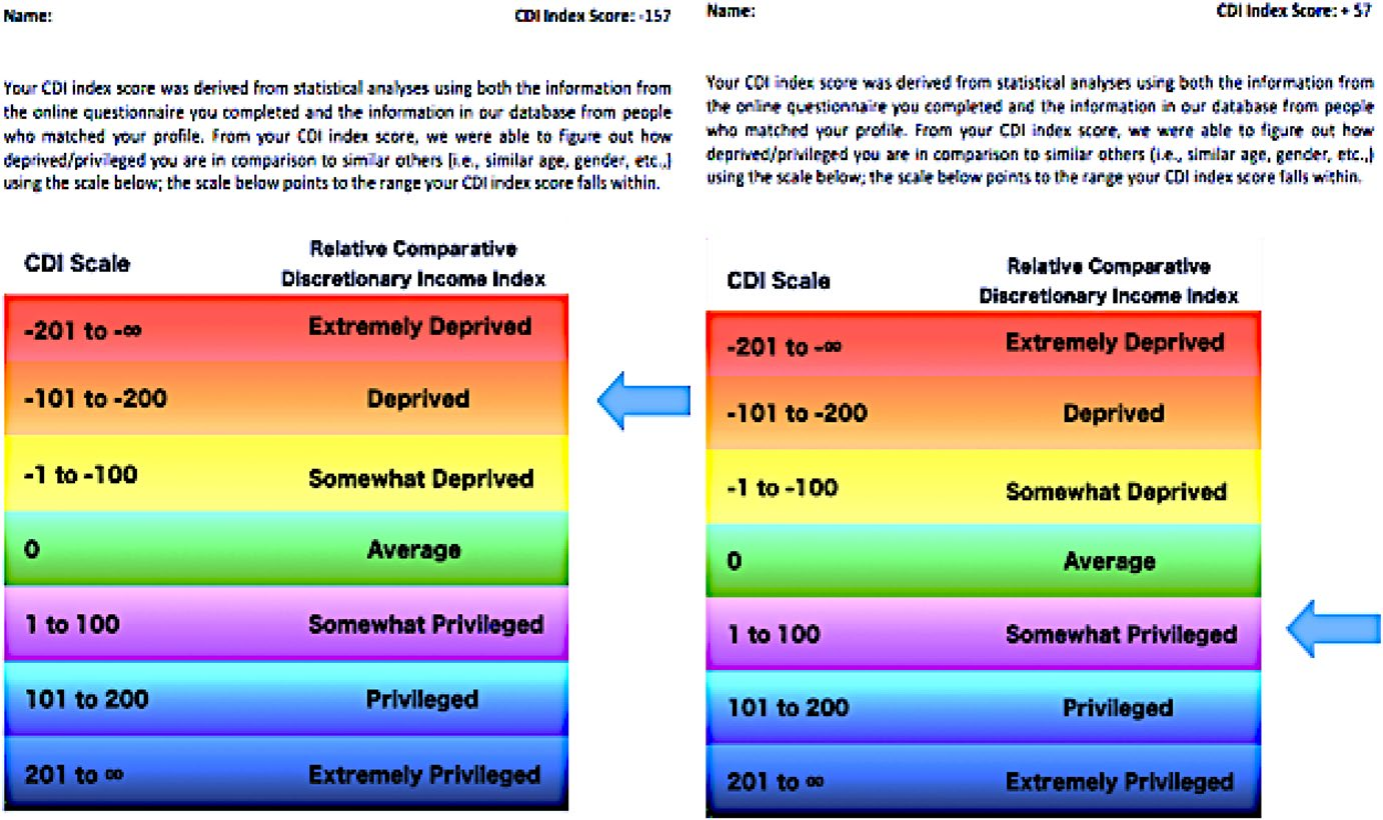
Experimental manipulations: inducing feelings of relative deprivation/privilege

Once the text was read out to the participant, they were informed of their CDI Index Score and where on a scale of extremely deprived to extremely privileged, their score was located (participants were unaware that this scale was invented for this experiment and thus not a true scale); the page containing this information was then handed to the participants for review (see Fig. 4).

Once participants had reviewed the above page (Fig. 4), the procedure was the same as in Experiment 1.

#### Data Analyses

As in Experiment 1, a three-stage hierarchical regression model was used to analyze results; the outcome variable remained the same (*potential returns* across ten experimental rounds, labelled as *risk-taking*). In addition to age and gender, family income was also entered at stage one of the regression model. Agency-thinking, pathway-thinking and condition (categorical variable: relatively deprived vs. relatively privileged) were entered at stage two of the model. Finally, two interaction variables (Agency X Condition and Pathway X Condition) were computed and entered at stage three of the regression model. The rejection level for all analyses was set at *p* = 0.05.

### Results

**Fig. 5:**
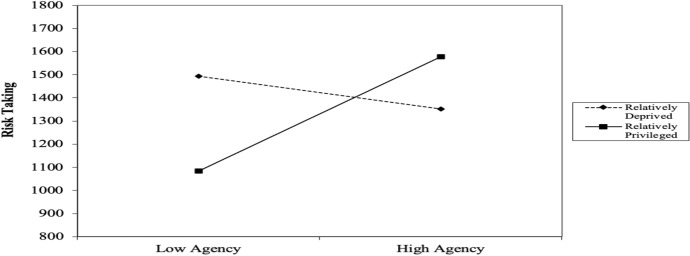
The effect of relative deprivation/privilege (experimentally induced) and agency-thinking (in interaction) on risk-taking in Experiment 2. Note. Points are plotted at ±1 SD of the mean

One participant failed the task-comprehension questionnaire and was excluded from analyses. The remaining 50 (40 females and 10 males) participants’ data were used for analyses; participants’ age ranged from 18 to 39 years old (*M* = 20.34, *SD* = 3.31). A three-stage hierarchical regression was conducted to test whether feelings of relative deprivation/privilege caused changes in risk-taking among low and high-hope individuals (see Table 3 for regression statistics). The results indicated that among relatively privileged persons, as agency-thinking increases, so does risk-taking, whereas, among the relatively deprived, as agency-thinking increases, risk-taking reduces (see Fig. 5).

**Table 3:** Summary of hierarchical regression analysis for variables predicting risk-taking

Variable	*β*	*95% CI (LL, UL)*	*t*	*SE*	*R*	*R*^*2*^	*ΔR*^*2*^	*F*	*ΔF*	*ΔF p*	*p*
**Model 1**					**0.24**	**0.06**	**0.06**	**0.96**	**0.96**	**0.419**	**0.419**
Age	0.04	(−19.57, 25.93)	0.28	11.30							0.780
Gender	− 0.18	(−304.01, 68.17)	− 1.28	92.45							0.209
Family income	− 0.18	(−30.47, 7.42)	− 1.22	9.41							0.227
**Model 2**					**0.25**	**0.06**	**0.00**	**0.47**	**0.03**	**0.993**	**0.830**
Age	0.05	(−20.96, 28.97)	0.32	12.38							0.748
Gender	− 0.19	(−319.45, 74.57)	− 1.25	97.69							0.217
Family income	− 0.19	(−33.76, 8.92)	− 1.17	10.58							0.247
Agency	0.06	(−26.18, 33.33)	0.24	14.75							0.810
Pathway	− 0.01	(−27.16, 26.24)	− 0.04	13.24							0.972
Condition	− 0.02	(−199.47, 183.53)	− 0.08	94.96							0.933
**Model 3**					**0.56**	**0.32**	**0.26**	**2.39**	**7.72**	0.001***	0.033*
Age	0.02	(−20.35, 24.16)	0.17	11.02							0.863
Gender	− 0.22	(−311.30, 35.04)	− 1.61	85.75							0.115
Family income	− 0.41	(−47.07, −6.39)	− 2.65	10.07							0.011*
Agency	− 0.24	(−43.99, 14.91)	− 1.00	14.58							0.325
Pathway	− 0.09	(−31.93, 20.48)	− 0.44	12.98							0.661
Condition	− 0.18	(−267.56, 84.55)	− 1.05	87.18							0.300
Agency X Condition	0.63	(11.19, 134.94)	2.39	30.64							0.022*
Pathway X Condition	0.18	(−36.56, 80.83)	0.76	29.06							0.451

*Note. N* = 50; *p < 0.05; **p < 0.01; ***p < 0.001 *LL* and *UL* indicate the lower and upper limit of a Confidence Interval (for *B)*; A post hoc power analysis indicated that our results produced a power of 90.9%, (*1-β err prob* = 0.909) indicating that this study had an adequate sample size

### Discussion

In Experiment 2, we found the same pattern of results as in Experiment 1, though by experimentally inducing feelings of relative deprivation/privilege, our results confirm that feelings of relative deprivation *cause* greater risk-taking among individuals with low agency, while higher agency ameliorates the damaging effect of RD. Therefore, across Experiments 1 and 2, we have shown that an interplay between hope and RD predict changes in risk-taking. More specifically, our findings indicate that hope (specifically, the agency component of hope) can buffer against risk-taking among the relatively deprived. Next, we wanted to examine whether a similar effect was found in a gambling population, thus testing whether these two constructs together have the predicted effect on a real-world risk behavior, problem gambling severity.

## Experiment 3

There is some evidence that people rely on excessive gambling to compensate for feelings of resentment stemming from RD (e.g., Callan et al. 2008, 2011). Studies have also shown that Problem Gambling often appears when individuals use gambling as a means of avoiding, coping with and/or escaping negative emotions (see Blaszczynski and Nower 2002). However, our findings from Experiments 1 and 2 provide some indication that hope can buffer against excessive gambling among individuals who view risk-taking (e.g., gambling) as a means of allaying negative feelings. Accordingly, we predict that among relatively deprived individuals, increases in hope will decrease gambling severity, as measured by the Problem Gambling Severity Index (PGSI: Ferris and Wynne 2001). Therefore, while Experiments 1 and 2 examined risk-taking in the laboratory, Experiment 3 aims to examine real-world risk behavior (gambling severity among gamblers).

### Methods

#### Participants

We were targeting a sample of at least 51 participants necessary to uncover a large effect size (*R*^2^ = 0.25) in a regression model which includes seven predictor variables, on the basis of an alpha of 0.05% and 80% power (*1-β err prob* = 0.8). However, to overcome low response rates common with Web surveys (see Couper 2000), we used snowball sampling to recruit participants over 20 days, which led to 236 participants starting the survey (advertised on Twitter), of which a total of 122 participants (52%) completed the survey and were included in analyses. While snowball sampling has some drawbacks (e.g., little control over the demographic/number of participants recruited), it is a useful approach for overcoming low response rates common with Web surveys and recruiting from hard-to-recruit populations (Berndt 2020; Sharma 2017). Indeed, the drawbacks of the employed snowball sampling method may be one key reason why the majority of the participants recruited were male (i.e., male participants may have engaged more with the study and in turn recruited more male friends to complete the study). Participants consisted of 17 females and 105 males, whose age ranged from 18 to 60 years old (*M* = 28.80, *SD* = 9.25). All 122 participants who completed the online questionnaire had gambled at least once in the past 12 months.

#### Materials and Procedure

A survey created on Qualtrics was advertised online (Twitter). The post included a link to the survey and an invitation letter that highlighted, to complete the questionnaire study, one must be 18 years of age or over and have gambled at least once in the past 12 months; these two conditions were again specified on the consent form. Moreover, a clear statement on the consent form emphasised that responses to this survey will be kept confidential and anonymous; empirical evidence indicates that individuals disclose sensitive information more accurately under anonymous conditions (see Ong and Weiss 2000; Werch 1990).

On the online survey, participants were asked to provide demographic information (age and gender) and whether they had gambled at least once in the past 12 months. Participants were then asked to complete The Adult Hope scale, the PRDS (both identical to Experiments 1) and the Problem Gambling Severity Index (PGSI).

##### Problem Gambling Severity Index (PGSI)

Ferris and Wynne’s (2001) 9-item PGSI (α = 0.94) is an assessment tool that was constructed to measure problem gambling severity in the general population. Each of the nine items (e.g., *when you gambled, did you go back another day to win back the money you lost?*) require respondents to answer on a four-point likert scale (0 = never, 1 = sometimes, 2 = most of the time, 3 = almost always). The total score is interpreted as: no gambling problem (score of 0); low level of problems with few or no identified negative consequences (score of 1 or 2); moderate level of problems leading to some negative consequences (scores between 3 and 7); or problem gambling with negative consequences and a possible loss of control (scores of 8 or more).

Typically, absolute PGSI scores are converted into four categories (no-problem, low-risk, moderate-risk and problem gambling) and are treated as a continuous measure of problem gambling severity (see Currie et al. 2013). However, the numerical scoring system of the PGSI is more consistent with the characteristics of an ordinal scale than a ratio scale (i.e., the equivalency between scale points is not assumed). Therefore, the range of scores for each category varies considerably; a 1-point range is used for the non-problem gambling category, a 2-point range for the low-risk, a 5-point range for the moderate-risk and a 19-point range for the problem gambling category, which could impact the temporal stability of the PGSI classifications (Currie et al. 2013). Nevertheless, evidence suggests that the PGSI is psychometrically stronger than similar screening tools including the South Oaks Gambling Screen (SOGS), the Victorian Gambling Screen and DSM-IV based scales (McMillen and Wenzel 2006; Orford et al. 2010).

#### Data Analyses

As recommended, the outcome variable (PGSI) was converted to a categorical variable. The four categories included: non-problem (score of 0), low-level (scores of 1 or 2), moderate-level (scores between 3 and 7), or problem (scores of 8 or more) gambling (Ferris and Wynne 2001). A three-stage hierarchical regression model was used to examine whether the relation between RD and gambling severity was moderated by either the *agency-thinking* or *pathway-thinking* components of hope, when controlling for age and gender.^1^ The rejection level for all analyses was set at *p* = 0.05.

### Results

**Fig. 6:**
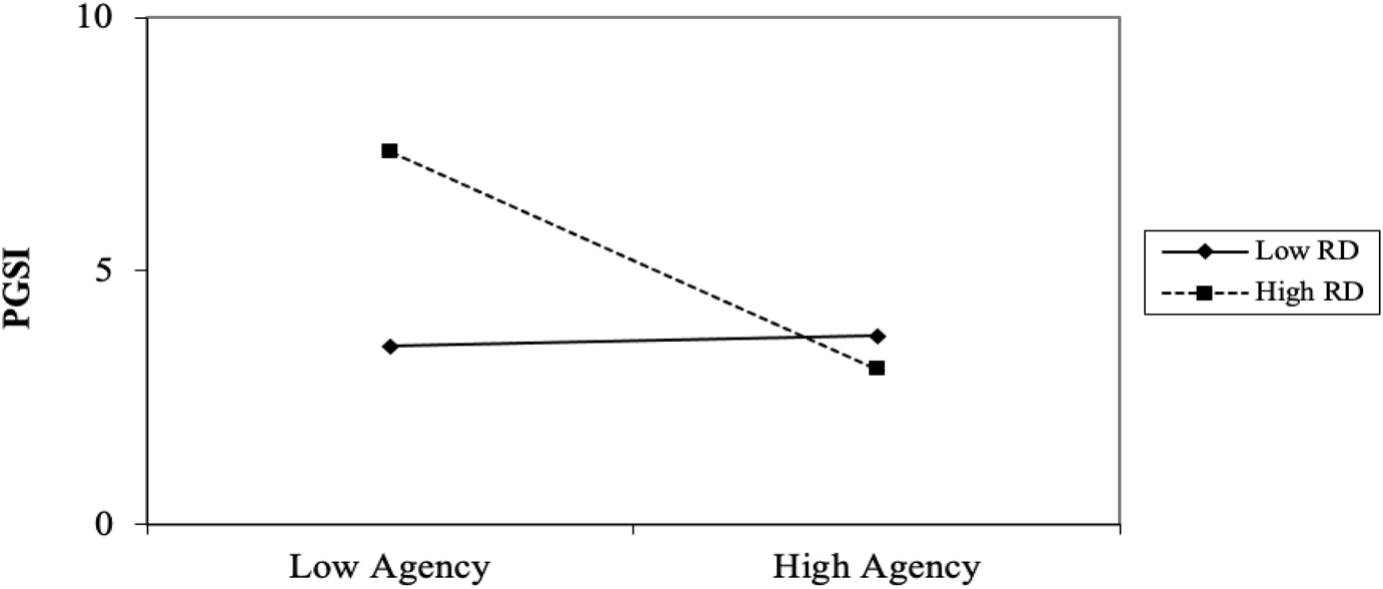
The effect of agency-thinking and RD (in Interaction) on PGSI in Experiment 3. Note. Points are plotted at ±1 SD of the mean

A total of 122 participants’ data were used for analyses: as identified by PGSI scores, 33 participants had no gambling problems (27%), 32 had low level of problems with few or no identified negative consequences (26%), 46 had moderate level of problems leading to some negative consequences (38%) while 11 participants were problem gamblers with negative consequences and a possible loss of control (9%). A three-stage hierarchical regression was conducted with categorized PGSI scores as the outcome variable (see footnote 1). The covariate variables age and gender were entered at stage one of the regression model. The predictor variables agency, pathways and RD were entered at stage two of the regression and two interaction variables (agency X RD and pathways X RD) were computed and entered at stage three. Significant results indicate that hope (agency component) plays a moderating role in the relation between RD and gambling severity (see Table 4 for regression statistics). Although categorized PGSI scores were used to run the regression analysis, absolute PGSI scores were used to visually demonstrate the relation between RD and gambling severity among low and high agency-thinking persons (see Fig. 6).

**Table 4:** Summary of hierarchical regression analysis for variables predicting gambling severity

Variable	*β*	*95% CI (LL, UL)*	*t*	*SE*	*R*	*R*^*2*^	*ΔR*^*2*^	*F*	*ΔF*	*ΔF p*	*p*
**Model 1**					**0.20**	**0.04**	**0.04**	**2.46**	**2.46**	**0.090**	**0.090**
Age	− 0.11	(− 0.03, 0.01)	− 1.17	0.01							0.245
Gender	− 0.17	(− 0.95, 0.04)	− 1.83	0.25							0.069
**Model 2**					**0.46**	**0.21**	**0.17**	**6.13**	**8.27**	0.001***	0.001***
Age	− 0.06	(− 0.02, 0.01)	− 0.77	0.01							0.445
Gender	− 0.13	(− 0.83, 0.11)	− 1.51	0.24							0.134
Agency	− 0.28	(− 0.10, − 0.02)	− 2.69	0.02							0.008**
Pathways	− 0.07	(− 0.07, 0.03)	− 0.63	0.03							0.528
RD	0.17	(− 0.01, 0.08)	1.86	0.02							0.065
**Model 3**					**0.49**	**0.24**	**0.04**	**5.27**	**2.68**	**0.073**	0.001***
Age	− 0.07	(− 0.02, 0.01)	− 0.82	0.01							0.412
Gender	− 0.12	(− 0.79, 0.14)	− 1.37	0.24							0.173
Agency	− 0.30	(− 0.11, − 0.02)	− 2.92	0.02							0.004**
Pathways	− 0.05	(− 0.06, 0.04)	− 0.47	0.03							0.643
RD	0.15	(− 0.01, 0.07)	1.70	0.02							0.091
Agency X RD	− 0.24	(− 0.02, − 0.01)	− 2.32	0.01							0.022*
Pathways X RD	0.14	(− 0.01, 0.02)	1.35	0.01							0.178

*Note. N* = 122; *p < 0.05; **p < 0.01; ***p < 0.001; *LL* and *UL* indicate the lower and upper limit of a Confidence Interval (for *B*); A post hoc power analysis indicated that our results produced a power of 99.8%, (*1-β err prob* = 0.998) indicating that this study had an adequate sample size

### Discussion

Consistent with our predictions, an inverse correlation between hope (agency component) and problem gambling severity existed among relatively deprived individuals, indicating that high agency-thinking individuals are less likely to gamble problematically in the face of RD. However, our results indicate that there is no significant relation between hope (either component) and gambling severity among relatively privileged persons. In sum, findings from this experiment support the notion that hope—specifically the agency component of hope—can act as a buffer against excessive gambling among the relatively deprived, a population known to participate in a range of maladaptive behaviors to cope with feelings of resentment stemming from RD (see Smith et al. 2012).

## General Discussion

While there is evidence that some RD individuals engage in maladaptive activities (i.e., gambling) to temporarily alleviate negative feelings stemming from RD (Callan et al. 2008), not all RD individuals engage in maladaptive behaviors (Zoogah 2010). This paper posited that hope—a unique form of positivity that looms during negative circumstances to help improve the future—would predict whether individuals engage in maladaptive behaviors to cope with RD or not. Consistent with our predictions, in Experiment 1, we found an inverse correlation between agency-thinking (i.e., goal-directed energy) and risk-taking among the relatively deprived. Employing an experimental design, Experiment 2 demonstrated that feelings of relative deprivation *cause* greater risk-taking among low agency-thinking individuals, providing further confirmation that hope—specifically the agency component of hope—can buffer against excessive risk-taking among the relatively deprived. Building on the first two experiments, results from Experiment 3 showed that hope and RD have the predicted association on real-world risk behavior (gambling behavior). While Snyder et al. (1991) suggested that both components of hope are fundamental in goal pursuit, our results indicate that only agency-thinking impacts risk-taking. These findings are consistent with Snyder’s (1994, 2002) view that agency-thinking is especially vital when encountering difficulties. Taking our findings into account, we encourage future research to examine whether factors that enrich agency-thinking are particularly useful in protecting at-risk individuals from participating in illicit behaviors.

Although this paper’s primary focus was to explore the impact of relative deprivation on risk behavior, our findings that relatively affluent individuals take greater risks when high in hope (as found in Experiments 1 and 2) requires further interpretation. It is the case that affluent individuals also engage in illicit behaviors (e.g., Racz et al. 2011), but we speculate that unlike their relatively deprived counterparts, their desire to engage in maladaptive behaviors does not root from their need to allay negative feelings. Instead, the relatively affluent engage in illicit behaviors for recreational purposes (see Sterk-Elifson 1996; see also Blaszczynski and Nower 2002 for discussion of multiple pathways to problem gambling). Thus, characteristics of hope, such as the enhanced ability to cope with negative events, may cause the relatively affluent to appraise risks more positively (i.e., not ruminate on potential adverse consequences), in turn encouraging these individuals to take greater risks. However, as the relatively affluent participate in maladaptive behaviors for recreational purposes, they are likely to stop when the fun stops, which would suggest that hope would not influence their real-world risk behavior (as found in Experiment 3). Although findings across this paper are supportive of our interpretation, further research is required to confirm these claims.

This paper has some limitations. It is important to recognise that the majority of participants in the first two experiments were female psychology undergraduates, thus it is unclear whether (or not) the findings from these experiments can be generalised to the general population. Similarly, the use of snowball sampling in Experiment 3 meant that we were unable to recruit a similar number of female and male gamblers, thus our findings should be interpreted with some caution. Finally, due to budgetary/financial constraints, not all participants in Experiments 1 and 2 received money for points gained during the risk game, though empirical evidence indicates that such a payment structure does not impact behavior (see Charness et al. 2016).

Despite these limitations, our findings across all three experiments indicate that hope can protect the relatively deprived from engaging in risky behaviors. More specifically, our findings that agency-thinking reduces risk-taking and gambling severity among a relatively deprived population (as demonstrated across three experiments) can have real-world implications and thus merits further discussion, especially as our results suggest that hope may make for positive interventions in cases of problem gambling. To conclude, we believe that our findings should encourage scholars to conduct intervention-based studies to examine whether hope can be used as an intervention in cases of problem gambling and similar maladaptive behaviors.

## Data Availability

Materials used across Experiments 1 to 3 are either (a) referenced and can thus be accessed from original sources, (b) available on the Open Science Framework (osf.io/dy7cg/), or (c) explained in detail so that researchers are able to reproduce similar. Moreover, data used for analyses across all three experiments are available on the same Open Science Framework repository.

## Footnotes

1 Although it is suggested that the PGSI scale—once converted into categories of non-problem, low-risk, moderate-risk, and problem gambling—should be treated as a continuous scale, it remains a concern that the scale shares more characteristics with an ordinal scale than a ratio scale. As such, we conducted an ordinal regression (all variables remained the same) and a multiple linear regression that included all the same predictor variables but substituted the outcome variable (from the typical categorical processing of the PGSI) to absolute PGSI scores. Results from both the ordinal regression [*X*^*2*^ (7, *N* = 122) = 33.41, *p* < 0.001, *Nagelkerke R*^*2*^ = 0.26] and the multiple linear regression with absolute PGSI entered as the outcome variable [*F*(7, 114) = 5.92, *p* < 0.001, *R*^*2*^ = 0.27] reveal the same pattern of significant results (i.e., model and Agency X RD interaction term are significant).
